# Intrinsic Frequencies of the Resting-State fMRI Signal: The Frequency Dependence of Functional Connectivity and the Effect of Mode Mixing

**DOI:** 10.3389/fnins.2019.00900

**Published:** 2019-09-04

**Authors:** Nicole H. Yuen, Nathaniel Osachoff, J. Jean Chen

**Affiliations:** ^1^Rotman Research Institute at Baycrest, Toronto, ON, Canada; ^2^Department of Medical Biophysics, University of Toronto, Toronto, ON, Canada

**Keywords:** resting-state fMRI, resting state functional connectivity, intrinsic mode function, frequency dependence characteristics, variational modal decomposition, empirical mode decomposed, physiological origins

## Abstract

The frequency characteristics of the resting-state BOLD fMRI (rs-fMRI) signal are of increasing scientific interest, as we discover more frequency-specific biological interpretations. In this work, we use variational mode decomposition (VMD) to precisely decompose the rs-fMRI time series into its intrinsic mode functions (IMFs) in a data-driven manner. The accuracy of the VMD decomposition of constituent IMFs is verified through simulations, with higher reconstruction accuracy and much-reduced mode mixing relative to previous methods. Furthermore, we examine the relative contribution of the VMD-derived modes (frequencies) to the rs-fMRI signal as well as functional connectivity measurements. Our primary findings are: (1) The rs-fMRI signal within the 0.01–0.25 Hz range can be consistently characterized by four intrinsic frequency clusters, centered at 0.028 Hz (IMF4), 0.080 Hz (IMF3), 0.15 Hz (IMF2) and 0.22 Hz (IMF1); (2) these frequency clusters were highly reproducible, and independent of rs-fMRI data sampling rate; (3) not all frequencies were associated with equivalent network topology, in contrast to previous findings. In fact, while IMF4 is most likely associated with physiological fluctuations due to respiration and pulse, IMF3 is most likely associated with metabolic processes, and IMF2 with vasomotor activity. Both IMF3 and IMF4 could produce the brain-network topology typically observed in fMRI, whereas IMF1 and IMF2 could not. These findings provide initial evidence of feasibility in decomposing the rs-fMRI signal into its intrinsic oscillatory frequencies in a reproducible manner.

## Introduction

The frequency characteristics of the resting-state BOLD fMRI (rs-fMRI) signal are of increasing scientific interest (Salvador et al., 2005; Niazy et al., 2011; Kalcher et al., 2014), as we discover more frequency-specific biological interpretations within the conventional data-acquisition bandwidth of 0–0.25 Hz (Golestani et al., 2015; Hocke et al., 2016). In particular, specific spectral content have been associated with physiological and vascular processes (Birn et al., 2008; Golestani et al., 2015; Mark et al., 2015; Hocke et al., 2016) and with the resulting brain-network measures (Nikolaou et al., 2016). Thus, spectral analysis of the rs-fMRI signal appears to be a compelling approach to achieving a better appreciation of how much neurally relevant information is conveyed by rs-fMRI. To that end, band-pass filtering has been used in the initial efforts (Kalcher et al., 2014). However, in theory, the rs-fMRI signal does not lend itself to conventional band-pass filtering approaches, as it is not stationary and cannot be defined by a few frequencies in Fourier domain. The band-pass filtering approach is inadequate for studying non-stationary signals, as the frequency content of such signals changes with time, while a filter bank is limited by assumptions regarding frequency, bandwidths and the type of filter design.

Previous studies examining the frequency characteristics of the resting-state BOLD fMRI (rs-fMRI) signal were largely based on the decomposition of the rs-fMRI signal into its intrinsic mode functions (IMFs). Existing studies (Niazy et al., 2011; Song et al., 2014) have used empirical mode decomposition (EMD) (Huang et al., 1998) and later used complementary ensemble empirical mode decomposition (CEEMD) (Wu and Huang, 2009; Yeh et al., 2012; Qian et al., 2015). At the core of EMD and CEEMD is a simple workflow. Signal local minima and maxima are recursively detected, based on which an upper and lower envelope are obtained through interpolation; subsequently, the midline of the resultant envelope is removed and the high-frequency component becomes the IMF. The same process is then performed on the signal composed of the low-frequency midline. Thus, each IMF can be expressed as a combination of a low-frequency amplitude-modulated and a high-frequency frequency-modulated signal Eq. (1),

(1) m⁢(t)=A⁢(t)⁢c⁢o⁢s⁢(∅⁢(t))

Where ∅(*t*) modulates the carrier frequency. The total bandwidth of this signal is described by Eq. (2),

(2) $$B\,W_{\text{IMF}}=2\,\left(\Delta \,f+f_{\text{FM}}+f_{\text{AM}}\right)$$

where Δ*f* is the total deviation from the instantaneous frequency of the IMF, while *f*_FM_ represents the maximum rate of the change of the instantaneous frequency, and *f*_AM_ represent the highest frequency of the envelope modulating the frequency-modulated signal. The IMF frequency estimate could be dominated by either of these, depending on noise conditions.

Previous works using EMD and CEEMD have both found that the rs-fMRI can be approximated by 4 to 5 IMFs covering the entire sampling bandwidth (Niazy et al., 2011; Qian et al., 2015), and that all IMFs can be used to reproduce similar network topologies. A fundamental assumption of EMD and its derivatives is that each IMF occupies a well-defined frequency range (Huang et al., 1998). In theory, the IMF-based representation is insensitive to non-stationarity and non-linearity in the original signal. EMD-type approaches are known to have difficulty separating tones of similar frequencies. Moreover, high levels of non-white noise can interfere with the accurate identification of the instantaneous frequency, and cause the frequency to appear to shift in a non-linear fashion, leading to mode mixing between IMFs (Wu and Huang, 2009) as well as the same mode to be spread across multiple IMFs. In addition, EMD-derived methods have a tendency to attribute wider bandwidths to IMFs occupying higher frequencies. This is likely a result of the recursions, which present the highest degree of uncertainty to the first (highest-frequency) IMFs, and not allowing for backward error correction after subsequent IMFs have been extracted. Indeed, when applied to rs-fMRI data, CEEMD resulted in visible modal widening as frequency increased (Qian et al., 2015).

Our study incorporates the usage of the recently proposed variational mode decomposition (VMD) method (Dragomiretskiy and Zosso, 2014) to decompose the BOLD rs-fMRI time series into its IMFs. Recently, the VMD method has recently found application in the analysis of geological signals (Liu et al., 2016; Xiao et al., 2016) and electrocardiographic data (Lahmiri, 2014; Mert, 2016; Tripathy et al., 2016). The theory of VMD has been described in detail elsewhere (Dragomiretskiy and Zosso, 2014), and will not be repeated here. We will simply point out that unlike its predecessors, VMD is non-recursive, and can reconstruct all modes simultaneously, controlled by a convergence criterion. The variational model assesses the bandwidth of the modes by minimizing the Gaussian-regularized mean-square error between the signal and its representation as a series of Wiener filters, with the modal instantaneous frequency being determined as the center of mass of the power-spectral density function of each IMF. This approach increases the robustness of the model to estimation uncertainties. VMD provides error checking, as the VMD solution is be updated by minimizing the mean-squared residual of all IMFs against the estimate of any given IMF. Lastly, the VMD convergence depends on a series of iterative optimizations, during which the balance between overfitting and signal-estimation accuracy can be adjusted, for instance, based on *a priori* knowledge about the signal.

In this work, we hypothesize that VMD is able to more precisely extract frequency bands from the rs-fMRI signal, reducing the issue of mode mixing and mode spreading demonstrated in the previous work. To verify this hypothesis, we tested the EMD, CEEMD, and VMD techniques through a Monte Carlo simulation. Furthermore, although prior work (using EMD and CEEMD) have suggested that resting-state networks (RSNs) measured through rs-fMRI are frequency independent, we hypothesize that existing results are affected by modal mixing and limited frequency precision as described herein. To address this hypothesis, we re-examine the frequency dependence of functional connectivity of RSNs using VMD.

## Materials and Methods

### VMD Optimization

As documented in the original paper, the VMD technique follows three steps: (1) estimate individual IMFs by computing the Hilbert transform of the original signal *f*; (2) shift each mode to its base frequency using heterodyne demodulation; (3) estimate the bandwidth of each mode as the H1 Gaussian smoothness of the demodulated signal. The target for the decomposition should be that

(3) $$\sum_{\text{k}}u_{\text{k}}=f$$

where *k* is the number of IMFs. If the signal *f* is smooth, the solution could be obtained through Tikhonov regularized minimization,

(4) $$m\,i\,n_{\text{f}}\,\left\{{||{\text{f-f}}_{0}||}_{2}^{2}+\alpha \,{||\partial_{\text{t}}f||}_{2}^{2}\right\}$$

where *f*_0_ is the measured signal, *f* is the original (clean) signal, and α is the regularization parameter. From this, we obtained the Euler-Lagrange equation:

(5) $$f-f_{0}=\alpha \,\partial_{\text{t}}^{2}f$$

where (*δ*_*t*_t is the partial derivative with respect to time and is the frequency in radians.

The minimization target of the VMD algorithm can be summarized by Eq. 6, which describes the inverse of the Gaussian smoothness of the demodulated signal,

(6) $$\underset{u_{k},w_{k}}{𝑚𝑖𝑛}\left\{||\partial_{t}\left[\left(\delta \left(t\right)+\frac{j}{\pi \,t}\right).u_{k}\left(t\right)\right]e^{-j\,w_{k}\,t}|{|}_{2}^{2}\right\}$$

where (δ(t) is Dirac’s delta function, $|{|}_{2}^{2}$ is the Euclidean norm squared, and j (=(v−1. This is solved using the augmented Lagrangian:

(7) $$ℒ\,\left(u_{k},w_{k},\lambda \right)=a+b+c$$

where

(8) $$a=\alpha \sum_{k}||\partial_{t}\left[\left(\delta \left(t\right)+\frac{j}{\pi \,t}\right).u_{k}\left(t\right)\right]e^{-j\,w_{k}\,t}|{|}_{2}^{2}$$

(9) $$b={||f-\sum_{\text{k}}u_{\text{k}}||}_{2}^{2}$$

(10) $$c=\left\langle \lambda ,f-\sum_{\text{k}}u_{\text{k}}\right\rangle$$

where (λ is the Langragian multiplier, b is the quadratic penalty term (squared residual) and c is the inner product of and the residual. The solution to the original variational problem is solved as the saddle point of the augmented Lagrangian. Each IMF is updated iteratively (by solving the VMD problem with respect to *u* and to ω until convergence is reached. In this way, all modes are extracted and optimized concurrently instead of sequentially.

In this study, we explicitly optimized the value of the regularization parameter α to balance the bandwidths of the spectral bands and the reconstruction error between the sum of the bands and the original signal. This parameter was chosen to minimize the overlap between the spectral bands in the Fourier domain while keeping the parameter as low as possible to retain reconstruction fidelity.

### Simulated Data

To compare the performance of the VMD method in relation to EMD and CEEMD methods, we performed a Monte Carlo simulation involving a known, “ground-truth” signal. First, we generated a signal composed of equal power contributions from four frequencies (0.03, 0.08, 0.15, and 0.23 Hz). This signal was sampled at 0. 25 Hz to emulate the typical sampling rate of rs-fMRI data (TR = 2 s), and the constituent frequencies were informed in part by those previously reported (Niazy et al., 2011; Qian et al., 2015). We then generated 200 variants of signal-noise mixtures, in which 200 different realizations of white noise time series were added to the signal to achieve a signal-to-noise ratio (SNR) of ∼1.2. This is representative of the lower end of the realistic SNR range in rs-fMRI data, particularly to accommodate the fact that the spectral signature of noise in real rs-fMRI data is imprecise and non-stationary. All three decomposition methods were then applied to extract the original frequencies. To quantify the performances of the different algorithms, we computed the fractional inter-modal overlap (mode mixing) for each, defined as the amount of spectral power in the neighboring IMFs as a fraction of the total spectral power of each “ground-truth” IMF.

### MRI Data Acquisition

MRI data were collected from 8 healthy adults (mean age 30 ± 6.7 years) on a 3T Siemens TIM Trio scanner and a 32-channel head coil. Specifically, whole-brain resting-state fMRI (rs-fMRI) data were acquired using single-shot gradient-echo EPI. The conventional-TR scans are later referred as “long-TR” scans: 26 slices, TR = 2 s, flip angle = 70°, FOV = 220 mm × 200 mm, voxel size = 3.4 mm × 3.4 mm × 4.6 mm in 240 frames. To enable assessment of reproducibility of our methods, the rs-fMRI scan was performed twice for each subject (two trials per subject) within the same session. On a subset of seven subjects, we also acquired rs-fMRI data using simultaneous multi-slice (SMS) acceleration on the gradient-echo EPI (Feinberg et al., 2010) (TR = 323 ms, TE = 30 ms, flip angle = 40°, 15 slices, 3.44 mm × 3.44 mm × mm, 2230 time points, acceleration factor = 3, phase encoding shift factor = 2, slices ascending). The brain coverage of these “short-TR” scans was matched to that of the “long-TR” scans, and we only used 1486 frames of the short-TR scans for comparison with the long-TR scan results. This would permit us to assess the dependence of our results to fMRI sampling rate. A 3D T1-weighted anatomical scan was acquired using MPRAGE, with resolution 1 × 1 × 1 mm, repetition time (TR) = 2400 ms, inversion time (TI) = 1000 ms, echo time (TE) = 2.43 ms, flip angle = 8, field of view × 256 × 256 mm (sagittal), matrix size = 256 × 256, 192 slices (ascending order), bandwidth = 180 Hz/pixel, and GRAPPA acceleration factor = 2.

### Image Preprocessing

The rs-fMRI data were preprocessed using FSL FEAT version 5.0.8 (Jenkinson et al., 2002). Functional data had the first 10 volumes removed and skull stripped using the Brain Extraction Tool (BET). Data were corrected for motion (reference being the middle frame of each data set) and slice time then band-pass filtered to be between 0.01 and 0.25 Hz (using fslmaths, which implements a Gaussian filter). The cut-off of 0.25 Hz was chosen to represent the maximum detectable frequency in typical rs-fMRI acquisitions (i.e., TR = 2 s). Similar to prior work of a similar nature (Niazy et al., 2011; Tong et al., 2011), we did not actively correct physiological artifacts. The VMD technique was then used to decompose the preprocessed BOLD signal, and the results were compared to those obtained using the EMD and CEEMD methods.

The T1 anatomical scans were used in defining noise regions of interest (ROIs) for further analysis. Specifically, we used FMRIB Automated Segmentation Tool (FAST) for segmentation of gray matter, white matter, and cerebrospinal fluid ROIs. The FSL-FAST segmentation routine is based on a Hidden Markov Random Field model that is optimized using the expectation-maximization algorithm (Zhang et al., 2001). The ROI masks are then aligned with the fMRI data using anatomical-to-fMRI transformation matrices determined using FSL Flirt (Jenkinson and Smith, 2001; Jenkinson et al., 2002).

Furthermore, we performed cortical-surface reconstruction using FreeSurfer^1^. The procedure includes removal of non-brain tissue using a hybrid watershed/surface deformation procedure (Segonne et al., 2004), automated transformation into the MNI152 standard space, intensity normalization (Sled et al., 1998), tessellation of the gray matter white matter boundary, automated topology correction (Segonne et al., 2007), and surface deformation following intensity gradients to optimally place the gray/white and gray/CSF borders at the location where the greatest shift in intensity defines the transition to the other tissue class (Fischl and Dale, 2000). The subsequent segmentation of the cortex and subcortical gray matter volumetric structures were performed for each subject based on probabilistic models of tissue magnetic resonance parameters and of anatomical locations (Fischl et al., 2004). The resultant cortical models permitted surface inflation (Fischl et al., 1999) and registration to a spherical atlas, whereby individual cortical folding patterns were used to match cortical geometry across subjects (Fischl et al., 1999).

### IMF Clustering and Spectral Analysis

VMD was used decompose each voxel in the rs-fMRI data into a specified number of IMFs. For each IMF in each voxel, the frequency associated with the center of mass of the power spectral density function of each IMF was used to define the dominant frequency of said IMF. After this procedure was repeated for each voxel, one challenge remained – as each voxel is associated with a slightly different set of IMF frequencies, it was difficult to identify any generalizable findings regarding frequency content. To overcome this, we identified the existence of whole brain “*IMF frequency clusters*” by plotting the histograms of IMF frequencies including all IMFs of all voxels of each tissue type. In plotting the histogram, each IMF is weighted by its normalized power contribution (normalized by total spectral power at each voxel).

We then modeled the peaks in the histograms as Gaussian functions (Qian et al., 2015) and identified the *widths of the IMF frequency clusters* as including 95% of the areas of the fitted Gaussians. Using these cluster definitions, we classified each IMF from each voxel as belonging to an IMF cluster (named IMF 1–4), each associated with a distinct frequency range. We repeated this for all eight subjects, and assessed the reproducibility of these frequency ranges in gray and white matter using the intra-class correlation coefficient (ICC). For this purpose, each IMF map was further masked to include only white or only gray matter (FSL 5.0.8).

In this work, in order to arrive at the best number of IMFs to use, we compared IMF- frequency clusters resulting from assuming 2 IMFs, 4 IMFs, 5 IMFs and 8 IMFs. Sample IMF histograms are shown in Appendix Figure A1. The comparison metrics are precision and reproducibility. The precision metrics include: (1) the group-wise standard deviation of the frequency-cluster locations; (2) the group-wise standard deviation of the frequency-cluster widths. The reproducibility metrics include: (1) the percentage of subjects manifesting a particular frequency cluster; (2) the correlation between frequency-cluster locations estimated from 2 runs of each subject. The results are detailed in the (Appendix Figure A2), and indicate the choice of 4 IMFs produced the most precise and reproducible frequency-cluster estimates. Note that there was no direct link between the number of IMFs targeted for at the VMD stage and the number of IMF frequency clusters detected at the clustering stage.

Note that for the short-TR data set, we first low-pass filtered the data at 0.25 Hz in order to emulate the sampling rate of conventional rs-fMRI. The main difference between the data acquired at 0.25 Hz and the filtered short-TR data is that the latter is associated with reduced aliasing in the 0–0.25 Hz range.

### Amplitude Analysis

For both long- and short-TR data sets, we also computed the *fractional IMF amplitude*. This is computed at each voxel as the fractional contribution of the spectral power of each IMF (as defined by its associated IMF cluster) to the total spectral power of all IMFs (as defined by the remaining IMF clusters). This parameter was defined to overcome the limitation that the raw spectral powers of IMFs from different subjects and different acquisitions are not directly comparable (Zou et al., 2008), given variability in factors such as scanner tuning and analog-to-digital conversion range. To demonstrate the spatial distribution of the fractional IMF amplitude, we transformed each subject’s fractional amplitude map into MNI152 space using FSL flirt (Jenkinson et al., 2002). The reference image for the registration was the middle frame of the original fMRI data, and the resulting transformation matrix was applied to the IMF amplitude and frequency maps. Subsequently, we overlaid the group-mean fractional amplitude map onto a cortical-surface model using FreeSurfer (Fischl et al., 1999).

### Functional Connectivity Matrices

As the fMRI data were registered with MNI152 space, we used the automated anatomical labeling (AAL) (Tzourio-Mazoyer et al., 2002)to divide the brain into 116 anatomical regions of interest (ROIs), including both the cortex and the cerebellum. These ROIs are listed in Table 1. For each subject, we averaged all IMFs within each IMF-cluster frequency range in each ROI. We then generated matrices of Pearson correlation coefficients between the IMF time series of all pairs of ROIs. These were then averaged across subjects to provide an overview of RSN organization.

**TABLE 1:** List of regions of interest (ROIs) used when computing correlation matrices.

**Index**	**Region of Interest**	**Index**	**Region of Interest**	**Index**	**Region of Interest**
1	Precentral_L	40	ParaHippocampal_R	79	Heschl_L
2	Precentral_R	41	Amygdala_L	80	Heschl_R
3	Frontal_Sup_L	42	Amygdala_R	81	Temporal_Sup_L
4	Frontal_Sup_R	43	Calcarine_L	82	Temporal_Sup_R
5	Frontal_Sup_Orb_L	44	Calcarine_R	83	Temporal_Pole_Sup_L
6	Frontal_Sup_Orb_R	45	Cuneus_L	84	Temporal_Pole_Sup_R
7	Frontal_Mid_L	46	Cuneus_R	85	Temporal_Mid_L
8	Frontal_Mid_R	47	Lingual_L	86	Temporal_Mid_R
9	Frontal_Mid_Orb_L	48	Lingual_R	87	Temporal_Pole_Mid_L
10	Frontal_Mid_Orb_R	49	Occipital_Sup_L	88	Temporal_Pole_Mid_R
11	Frontal_Inf_Oper_L	50	Occipital_Sup_R	89	Temporal_Inf_L
12	Frontal_Inf_Oper_R	51	Occipital_Mid_L	90	Temporal_Inf_R
13	Frontal_Inf_Tri_L	52	Occipital_Mid_R	91	Cerebellum_Crus1_L
14	Frontal_Inf_Tri_R	53	Occipital_Inf_L	92	Cerebellum_Crus1_R
15	Frontal_Inf_Orb_L	54	Occipital_Inf_R	93	Cerebellum_Crus2_L
16	Frontal_Inf_Orb_R	55	Fusiform_L	94	Cerebellum_Crus2_R
17	Rolandic_Oper_L	56	Fusiform_R	95	Cerebellum_3_L
18	Rolandic_Oper_R	57	Postcentral_L	96	Cerebellum_3_R
19	Supp_Motor_Area_L	58	Postcentral_R	97	Cerebellum_4_5_L
20	Supp_Motor_Area_R	59	Parietal_Sup_L	98	Cerebellum_4_5_R
21	Olfactory_L	60	Parietal_Sup_R	99	Cerebellum_6_L
22	Olfactory_R	61	Parietal_Inf_L	100	Cerebellum_6_R
23	Frontal_Sup_Medial_L	62	Parietal_Inf_R	101	Cerebellum_7b_L
24	Frontal_Sup_Medial_R	63	SupraMarginal_L	102	Cerebellum_7b_R
25	Frontal_Med_Orb_L	64	SupraMarginal_R	103	Cerebellum_8_L
26	Frontal_Med_Orb_R	65	Angular_L	104	Cerebellum_8_R
27	Rectus_L	66	Angular_R	105	Cerebellum_9_L
28	Rectus_R	67	Precuneus_L	106	Cerebellum_9_R
29	Insula_L	68	Precuneus_R	107	Cerebellum_10_L
30	Insula_R	69	Paracentral_Lobule_L	108	Cerebellum_10_R
31	Cingulum_Ant_L	70	Paracentral_Lobule_R	109	Vermis_1_2
32	Cingulum_Ant_R	71	Caudate_L	110	Vermis_3
33	Cingulum_Mid_L	72	Caudate_R	111	Vermis_4_5
34	Cingulum_Mid_R	73	Putamen_L	112	Vermis_6
35	Cingulum_Post_L	74	Putamen_R	113	Vermis_7
36	Cingulum_Post_R	75	Pallidum_L	114	Vermis_8
37	Hippocampus_L	76	Pallidum_R	115	Vermis_9
38	Hippocampus_R	77	Thalamus_L	116	Vermis_10
39	ParaHippocampal_L	78	Thalamus_R		

*Sup, superior; Mid, middle; Inf, inferior; L, left; R, right.*

For comparison with the literature, we created an additional set of correlation matrices using the band-pass filtered data (at 0.01–0.08 Hz) for each subject. This is the frequency range typical of rs-fMRI analyses. Furthermore, to help explain the spectral makeup of this reference correlation matrix, we also generated correlation matrices using signals band-pass filtered into the frequency ranges corresponding to the IMFs. These were also averaged across subjects.

### Statistical Comparisons

In this work, comparison between IMF clusters and tissue types is performed using the Student’s *t*-test, and linear correlation is used as the similarity index.

## Results

**FIGURE 1:**
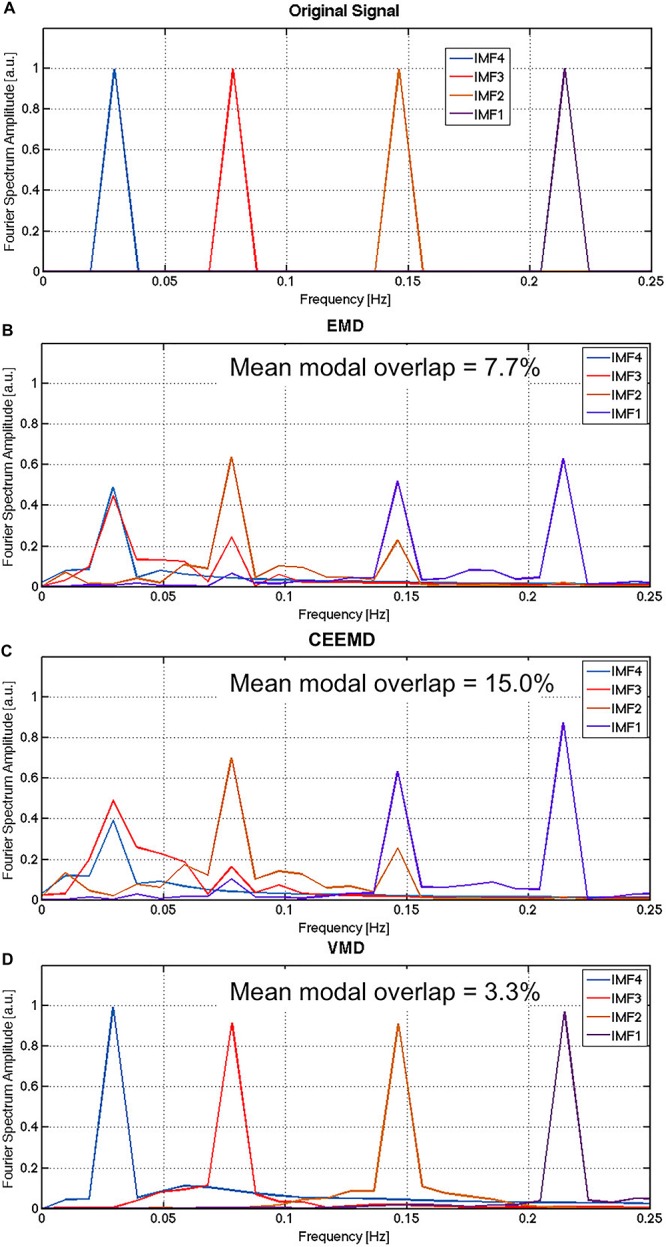
Comparison of performance for EMD, CEEMD and VMD on simulated data: The Fourier spectrum of the original noiseless signal **(A)** is compared to the average spectra obtained by EMD **(B)**, CEEMD **(C)** and VMD **(D)** on the noisy version if the signal, averaged across all iterations of the Monte Carlo simulation. Gaussian noise was simulated in this case, with the SNR of the simulated noisy signals was approximately 1.2, approximated by the total power of the noise over the total power of the signal. In EMD and CEEMD, the 4 IMFs with highest powers are displayed.

The average results of the Monte Carlo simulation are shown in Figure 1. While the noiseless signal was successfully reconstructed using all three algorithms (not shown), they performed very differently when noise was introduced. It is evident that IMF1 derived using EMD (Figure 1B) contains two distinct modes that would ideally have been attributed to two different IMFs. This is the manifestation of modal spreading, which is also seen in the CEEMD results (Figure 1C). Due to the existence of substantial modal spreading, inter-modal mixing (overlapping between IMFs in the frequency domain) is also found. Compared to EMD and CEEMD, VMD was able to identify the 4 IMFs of the original simulated signal with the least mode spreading and mode mixing (Figure 1D), with the noise component being split across the 4 IMFs. These simulation results confirm the theory-based hypothesis of increased IMF-estimation precision using VMD.

### Intrinsic Frequencies of the rs-fMRI Signal

**FIGURE 2:**
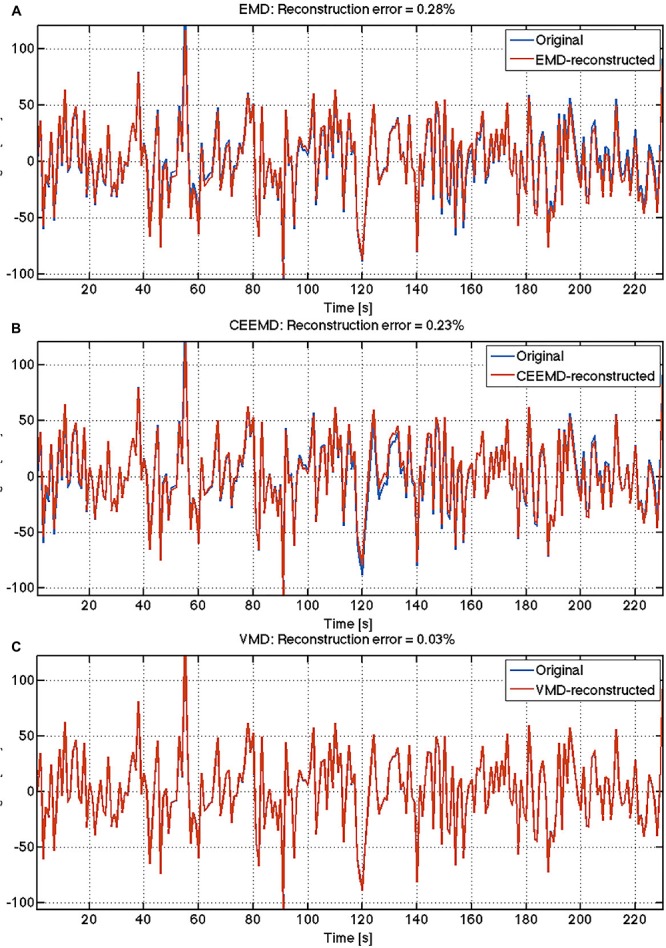
Comparison of reconstruction accuracy of rs-fMRI time series: The reconstruction errors are indicated for each algorithm: **(A)** EMD; **(B)** CEEMD; **(C)** VMD.

**FIGURE 3:**
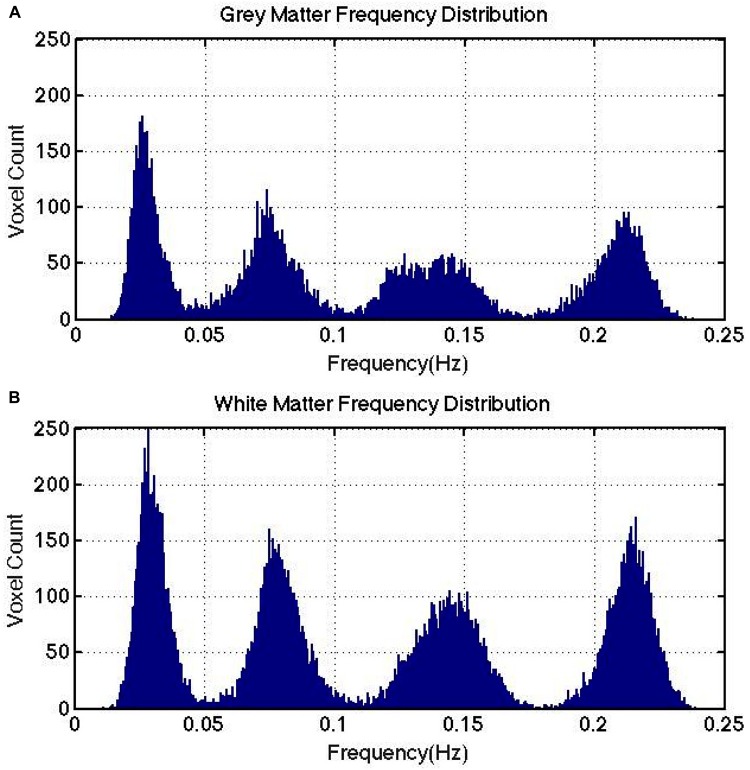
IMF frequency cluster distributions computed for a representative subject. Gray matter **(A)** and white matter **(B)** are shown to exhibit similar frequency clusters. Note that there is no direct link between the number of IMFs targeted for at the VMD stage and the number of IMF frequency clusters detected at the clustering stage.

Sample results from human rs-fMRI data are shown in Figure 2. VMD was able to extract IMFs from all data sets with high consistency and the lowest reconstruction error compared to both EMD and CEEMD. Based on results similar to these, IMFs were estimated for all voxels for each rs-fMRI data set. The corresponding IMF frequencies were organized into histograms for gray and white matter. A sample histogram of IMF frequencies is shown in Figure 3. Note that not all voxels returned 4 IMFs. Yet, when aggregated, the histograms revealed 4 clusters of IMF frequencies. This was common across all subjects, and across tissue types. Based on these clusters, IMF frequency ranges were identified for each subject in the group. We noted regional variability in the location of IMF clusters, with IMF4 being the most stable across brain regions and IMF2 being the least (Appendix Figure A3).

**FIGURE 4:**
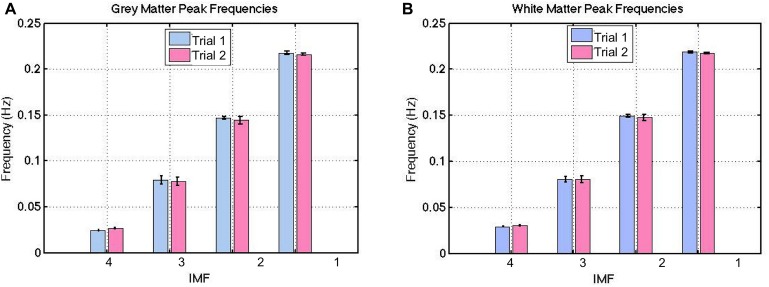
Between-session reproducibility of IMF frequency estimations. These frequencies were highly reproducible, with a ICC of 0.99 for both gray **(A)** and white matter **(B)**. The error bars indicate the standard deviations across all subjects.

The group-average VMD-decomposed peak frequencies in both gray and white matter are shown in Figure 4. Using the IMF-frequency clustering procedure described earlier, we identified 4 robust IMF-cluster frequency ranges. This was the case for all subjects and common to both gray and white matter. The frequency ranges of the 4 VMD IMF-frequency clusters are (mean frequency ± mean width/2):

• VMD IMF1: 0.20–0.24 Hz
• VMD IMF2: 0.13–0.17 Hz
• VMD IMF3: 0.063–0.098 Hz
• VMD IMF4: 0.021–0.036 Hz

These frequencies were evenly distributed across the majority of voxels (both gray and white matter), with no specific spatial features. Thus, we do not show spatial maps of the frequency distributions. Also, these frequencies were highly reproducible based on the long-TR acquisitions (Figure 4), with an ICC of 0.99 for both gray and white matter. This was confirmed by the absence of significant difference between IMF peak frequencies across each trial in either tissue type (*p* > 0.21). Henceforth, all IMFs associated with actual rs-fMRI data will be identified by their cluster numbers (i.e., IMF1-4 refer to IMF cluster 1–4).

**FIGURE 5:**
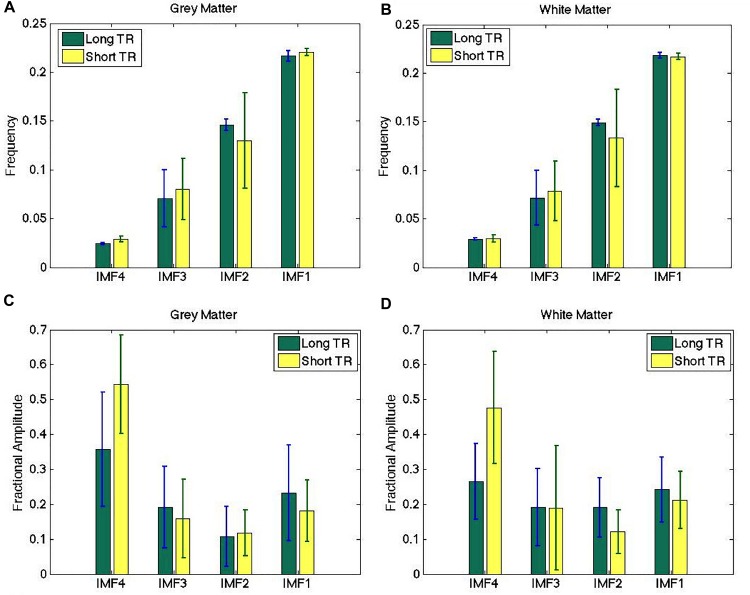
Comparison of IMF frequency **(A,B)** and fractional amplitude estimates **(C,D)** across different sampling rates. The IMF frequency groupings obtained from long-TR and short-TR data acquisitions are highly similar, although the fractional IMF amplitudes are less consistent across different TRs. There are no significant differences between the estimates obtained from the two data sets. The error bars indicate the standard deviations across all subjects.

The IMF frequency clusters obtained from long-TR and short-TR data acquisitions are highly similar, as shown by the frequency groupings identified in Figures 5A,B – at a group level, there were no significant differences between the two TRs for any of the IMFs. However, the fractional IMF amplitudes are less consistent across different TRs, as shown in Figures 5C,D. In particular, IMF4 (the frequency cluster with the lowest mean frequency) is a consistently greater contributor to total spectral power in short-TR data sets. However, once again, the differences are not statistically significant.

**FIGURE 6:**
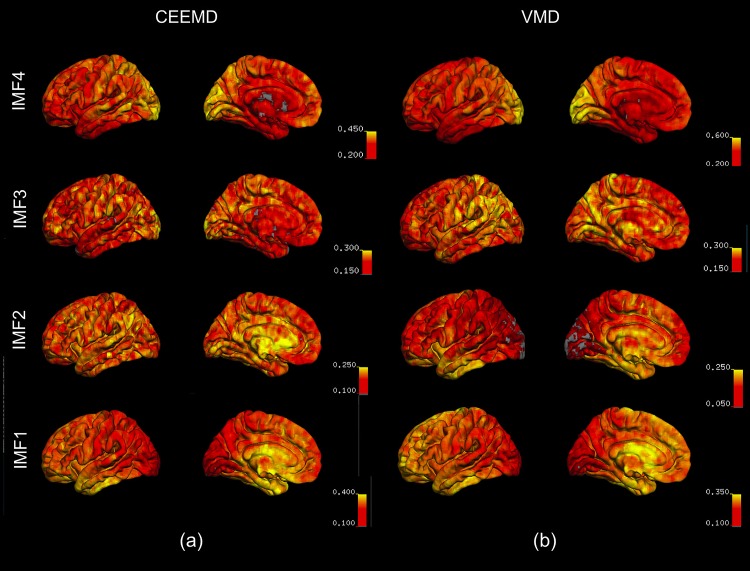
Spatial distribution of the fractional contributions of each IMF to the total spectral power of the rs-fMRI signal. The maps were averaged across all subjects for each IMF, and overlaid on a cortical surface. IMF4, which is associated with the lowest frequency, is markedly elevated in the occipital lobe, as reflected by VMD results **(b)**. This is consistent with the CEEMD results **(a)**. IMF1 and 2, associated with the highest frequencies, are elevated in the temporal lobe and the frontal/limbic cortices, respectively. These are not clearly seen in CEEMD-derived IMFs.

In Figure 6, we show the spatial distribution of the fractional contributions of each VMD-derived IMF to the total spectral power of the rs-fMRI signal. The equivalent maps are shown for CEEMD-based IMFs as well. Note that for CEEMD as well, 4 IMF clusters were identified within the range of 0.01–0.25 Hz, confirming previous findings by Qian et al. (2015). However, due to the aforementioned decreasing spectral resolution with increasing IMF frequency exhibited by CEEMD, the frequency ranges of the CEEMD IMF clusters, listed below, are not directly comparable to those of VMD.

• CEEMD IMF1: 0.12–0.23 Hz
• CEEMD IMF2: 0.05–0.12 Hz
• CEEMD IMF3: 0.025–0.05 Hz
• CEEMD IMF4: 0.01–0.025 Hz

For both VMD and CEEMD results, maps of fractional spectral power were averaged across all subjects for each IMF, and overlaid on a cortical surface. IMF4, which is associated with the lowest frequency, is markedly elevated in the occipital lobe, as reflected by VMD results (Figure 6b). This is consistent with the CEEMD results (Figure 6a). IMF1 and 2, associated with the highest frequencies, were elevated in the temporal lobe and the frontal/limbic cortices, respectively. These are not clearly seen in CEEMD-derived IMFs. It is important to note that the CEEMD-derived IMF frequency ranges were as follows: IMF1: 0.12–0.23 Hz, IMF2: 0.05–0.10 Hz, IMF3: 0.025–0.05 Hz, IMF4: 0.01–0.025 Hz. These 4 IMF groupings were chosen to best match those of the VMD groupings.

### Frequency Dependence of Network Organization

**FIGURE 7:**
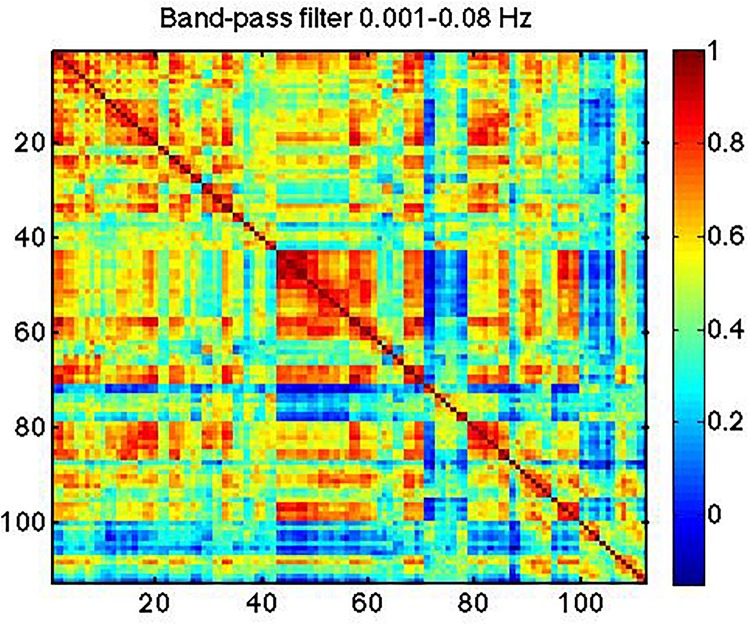
Typical correlation matrix based on the rs-fMRI signal within 0.001–0.08 Hz.

In Figure 7, we show strong RSN correlation patterns in areas that are part of the motor and control networks (indices 1–20), visual network (indices 43–60) and the medial-temporal network (indices 80–90). These results, based on the conventionally band-pass filtered rs-fMRI signal (to 0.01–0.08 Hz), are consistent with existing literature (Zhang and Li, 2014; Qian et al., 2015).

**FIGURE 8:**
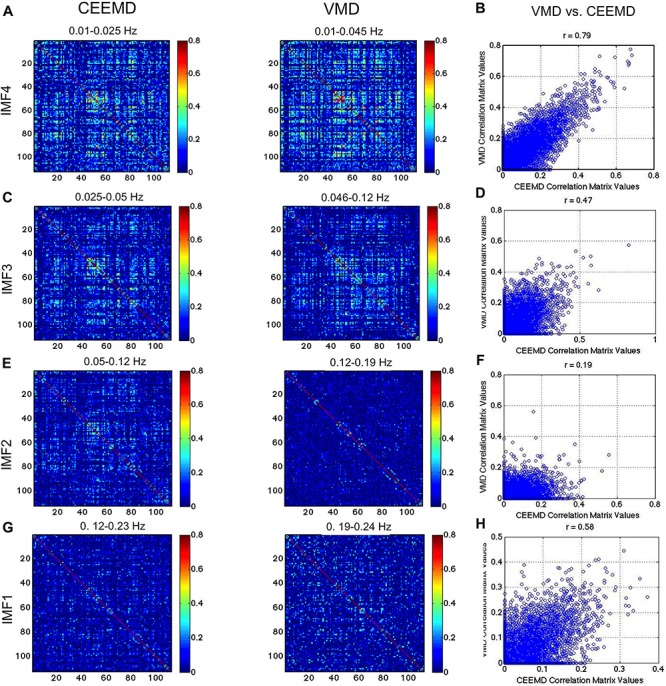
Comparison of VMD with CEEMD RSN-matrix topology. IMFs 4, 3, 2, and 1 are represented in **(A)**, **(B)**, **(C)**, and **(D)**, respectively. The VMD and CEEMD IMFs are in comparable frequency ranges. The highest agreement between VMD and CEEMD results are seen in IMF 4 (*r* = 0.79), and secondarily in IMF 1 (*r* = 0.58), the lowest and highest frequencies, respectively. Note that the maximum displayed correlations value has been reduced from 1 to 0.8 for display purposes.

In Figure 8, we compare the RSN topology derived from VMD with those based on the 4 IMFs in comparable frequency ranges obtained using CEEMD. We can see that the highest correlations are found using IMF4, followed by IMF3, while no strong patterns were seen for IMFs 1 and 2. Moreover, IMFs 3 and 4 were associated with correlation matrices that most resemble that of the conventionally band-pass filtered signal (Figure 7), but not in IMF 1 and 2. Even in IMFs 3 and 4, the values of the correlations are much lower than those from band-pass filtering. However, the values are comparable to those obtained based on CEEMD. In fact, we note that the highest agreement between VMD and CEEMD results can be seen in IMF 4 (Figure 8B, *r* = 0.79), and secondarily in IMF 1 (Figure 8H, *r* = 0.58), the lowest and highest frequencies, respectively.

**FIGURE 9:**
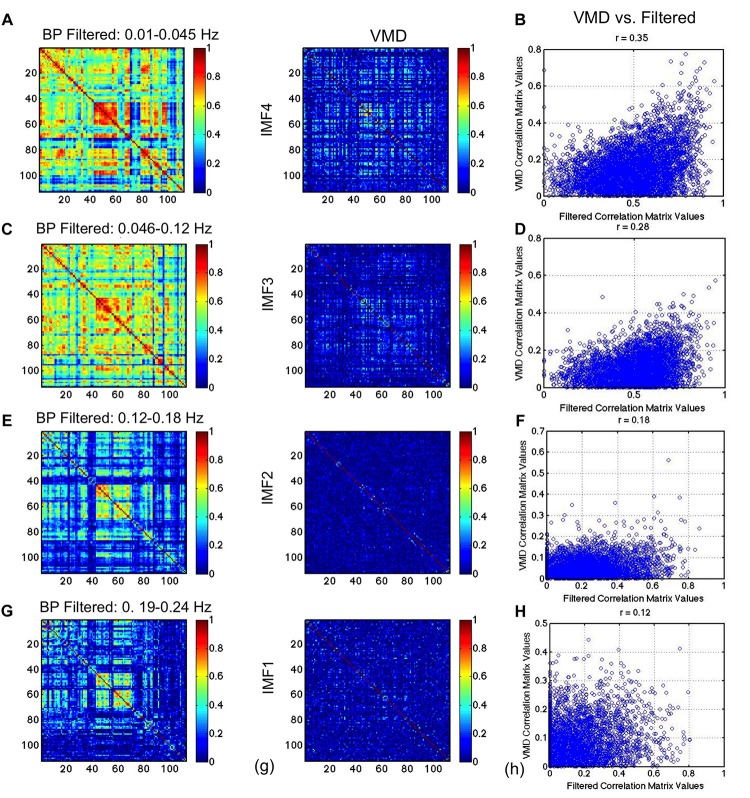
Comparison of VMD RSN-matrix topology with results of band-pass filtering. IMFs 4, 3, 2, and 1 are represented in **(A)**, **(B)**, **(C)**, and **(D)**, respectively. Each band-pass filter range determined based on VMD derivations of IMF frequency ranges. The conventional RSN topology can be observed across all band-passed frequencies, although it is most prominent at the lowest frequency. While this trend of decreasing correlation with increasing frequency is consistent with findings from VMD, the strengths of the correlations in VMD are markedly lower than those found using band-pass filtering.

In Figure 9, we repeat the comparison, substituting CEEMD with band-pass filtered versions of the rs-fMRI data, with each band-pass filter range determined based on VMD derivations of IMF frequency ranges. The conventional RSN topology can be observed across all band-passed frequencies (the top-left, middle and bottom-right areas of the matrix corresponding to the sensorimotor, visual and default-mode network, respectively), although it is most prominent at the lowest frequency. While this trend of decreasing correlation with increasing frequency is consistent with findings from VMD, the strengths of the correlations in VMD are markedly lower than those found using band-pass filtering. We also notice that the similarities (Figures 9B,D,F,H) are lower than observed between VMD and CEEMD results.

## Discussion

In studying the spectral properties of the rs-fMRI signal, there is an increasing desire to use data-driven methods rather than band-pass filtering to decipher frequency content (Niazy et al., 2011; Qian et al., 2015). The main differences between methods based intrinsic-modal decomposition and those based on band-pass filtering are: (1) results and interpretations of band-pass filtering are sensitive to the shape of the band-pass filter; (2) band-pass filtering is sensitive to non-linearity in the signal, such as introduced when the signal is non-stationary. The latter is particularly the case in rs-fMRI, affecting the oscillatory validity of band-pass filtered rs-fMRI signal components.

Our work is novel in the following aspects: (1) instead of relying on the assumption that our data-driven method accurately decomposes the modes of the signal, we test this accuracy using rs-fMRI-informed simulations; (2) we not only compared the findings from short-TR acquisitions with those based on conventional acquisitions (TR = 2 s), but also assessed the reproducibility of these results for both scenarios; (3) instead of defining the rs-fMRI spectral information by broad frequency ranges (up to >1 Hz), we specifically target the frequency range typically used in functional-connectivity analyses (<0.25 Hz).

Our primary findings are: (1) the rs-fMRI signal within the 0.01–0.25 Hz range can be consistently characterized by four intrinsic modal clusters (frequency clusters), centered at 0.028, 0.080, 0.15, and 0.22 Hz, respectively; (2) these frequency clusters were highly reproducible, and independent of rs-fMRI data sampling rate; (3) not all frequencies were associated with equivalent RSN topology, in contrast to previous findings.

### Intrinsic Frequencies of the rs-fMRI Signal

In this work, we demonstrate that as expected, compared to the previously used EMD and CEEMD techniques, VMD resulted in less inter-modal mixing as well as minimal modal spreading (Figure 1). When applied to rs-fMRI data, we demonstrate high reconstruction accuracy when using VMD-derived IMFs relative to the alternatives (EMD and CEEMD). We also see that VMD is able to decompose fMRI signals in a reproducible manner, given the small variability associated with each IMF frequency across 8 subjects. Interestingly, we found that both gray and white matter of the healthy brain are characterized by IMF clusters centered at the frequencies of 0.028, 0.080, 0.15, and 0.23 Hz, identifiable in all of our subjects. While all brain voxels exhibited these frequencies, the contribution of each to the total signal power varied spatially (Figure 6). In the literature, a similar clustering of IMF frequencies across brain voxels has been observed previously using EMD (Song et al., 2014) and CEEMD (Qian et al., 2015), although the previously reported center frequencies were 0.02, 0.04, 0.08, and 0.17 Hz, respectively. This difference is likely driven by the higher degree of mode spreading in the higher-frequency IMFs that is inherent in CEEMD, as described in the sections “Introduction” and “Results.”

When we used short-TR acquisitions (TR on the scale of 300 ms) (Niazy et al., 2011; Kalcher et al., 2014) to reduce respiratory and cardiac aliasing in the frequency range of interest, our findings of these IMF cluster frequencies did not change. Moreover, we acquired two trials of rs-fMRI data per subject, within the same scan session. To our best knowledge, no previous study has examined the reproducibility of intrinsic mode functions derived from rs-fMRI data, nor did any study examine the TR sensitivity of the decompositions. The frequency clusters we identified were highly reproducible across fMRI trials, and insensitive to the TR used (Figure 5), strengthening our confidence in the potential biological relevance of our findings.

### Possible Interpretations of Intrinsic rs-fMRI Frequencies

Our current data do not permit us to conclusively pinpoint the physiological source(s) of these frequencies, although we may refer to independent evidence of physiological oscillatory signatures.

IMF4, being at the lowest frequency, contributed the most to the overall signal power. This is in general agreement with findings by Kalcher et al. (2014), who nonetheless examined a different set of frequency bands (i.e., < 0.1 Hz, 0.1–0.25 Hz, 0.25–0.75 Hz, and 0.75–1.4 Hz). It has been well established that within the 0.01–0.25 Hz frequency range, low-frequency cardiac-rate variations and respiratory-volume variations are observable near 0.01 Hz and 0.036 Hz in fMRI data, respectively. Therefore, it is probable that IMF4 (range: 0.021–0.031 Hz) is associated with these phenomena. Indeed, the high power contribution of IMF4 to the occipital region (Figure 6) is consistent with previous reports on the amplitude of rs-fMRI BOLD signal modulation by respiratory variability (Chang and Glover, 2009a; Tong et al., 2011; Golestani et al., 2015). Moreover, the contribution of IMF4 to the overall power is the only fraction that is lower in white matter than in gray matter (Figures 5C,D), in agreement with recent findings by Tong et al. (2016), although the difference is not statistically significant. However, the effects of these different physiological variances could not be distinguished from one another, potentially due to oscillatory variations across different tissue locations (Chang and Glover, 2009a; Golestani et al., 2015). Moreover, while we may expect the short-TR data to exhibit lower IMF4 amplitude than long-TR data due to the enhanced ability for physiological nose removal, it is unclear which IMF the physiological signal would be aliased into in the long-TR data. At the maximum sampling frequency of 0.25 Hz, such noise sources could equally alias into IMF3. Indeed, such is the case in the gray matter, albeit the difference between short- and long-TR not statistically significant.

The frequency ranges of IMF3 (0.063–0.098 Hz, peaking at 0.080 Hz) and IMF2 (0.13–0.17 Hz, peaking at 0.15 Hz) have both been associated with low-frequency vascular oscillations (Tong and Frederick, 2014; Hocke et al., 2016; Tong et al., 2016). A major source of these vascular oscillations is vasomotion (Intaglietta, 1990; Rivadulla et al., 2011). Vasomotion has long been observed in the BOLD signal (Kiviniemi et al., 2000; Cordes et al., 2001), and refers to a spontaneous oscillation in the diameter of primarily pre-capillary vessels (Cooper et al., 1966) that propagates through the entire vasculature but does not influence cognitive processes. Vasomotion is associated with oscillations in red blood-cell velocity (Biswal and Hudetz, 1996) and modulates local blood flow (Morita et al., 1994; Biswal and Hudetz, 1996; Aalkjaer et al., 2011). In particular, initially observed in superficial blood vessels at around 0.1 Hz (Mayhew et al., 1996; Meyer et al., 2003; Murphy et al., 2013), vasomotion’s main frequency signature has been consistent between the animal (Mayhew et al., 1996; Bernardi, 1997; Haddock et al., 2002; Meyer et al., 2003) and human subcutaneous endothelium (Kvernmo et al., 1999, 1998).

The origins of vasomotion observed in fMRI could be caused by oscillations in both vascular diameter (Intaglietta, 1990; Biswal and Hudetz, 1996) and blood oxygenation (Biswal and Hudetz, 1996; Nikulin et al., 2014). Until recently, there have not been fMRI-based measurements of vasomotion in the human brain. Rayshubskiy et al. (2014) were able to measure sinusoids at (∼0.1 Hz near using intraoperative optical intrinsic-signal imaging and preoperative fMRI near the same pial veins of awake humans. However, an added challenge of *in vivo* isolation of vasomotion is that vasomotion frequency may in fact be dependent on vascular size, increasing with decrease vessel size (Intaglietta, 1990; Harrison and Cai, 2003). This, coupled with the fact that frequencies below 0.1 Hz (clusters IMF3 and IMF4) have typically revealed robust brain-network patterns, suggests that the effects of vasomotion may be embodied in IMF2. In support of the closer neuronal relevance of IMF3, we note that the regions of the highest fractional power distribution by IMF3 are the superior parietal, posterior cingulate and precuneus regions (Figure 6), coinciding with regions of high resting neuronal activity determined using positron-emission tomography (Raichle, 2011).

At very high frequencies (IMF1), the fractional spectral power is second only to the contribution of IMF4 (very low frequencies), as shown in Figure 5. This frequency band (0.20–0.24 Hz, peaking at 0.21 Hz) has previously been associated with head motion (Razavi et al., 2008). Although motion was corrected in the preprocessing pipeline, the effect of motion cannot be completely removed (Faraji-Dana et al., 2016a, b). In the case of the long-TR data, this frequency may also be associated with aliased cardiac pulsations, although this theory is refuted by the fact that IMF1 is equally strong in short-TR and long-TR data sets. The functional significance of IMF1 will need to be interpreted in the context of network-related features.

### Frequency Dependence of Network Organization

We found that the functional connectivity patterns of RSNs are dependent on frequency and that not all IMFs reveal the same connectivity patterns, contrary to previous reports (Niazy et al., 2011; Qian et al., 2015). The connectivity-matrix patterns found in IMF 3 and 4 (Figure 7) were most similar to those from the 0.01–0.08 Hz band-passed signal (Figure 8), and were not seen in the higher frequency range (IMF 1 and 2). This is expected and is likely to reflect differences in biological significance of high- and low-frequency signals as described earlier.

Our findings echo those of Song et al. (2014), who found cortical RSNs to be best represented in low-frequency oscillations (<0.05 Hz). While both IMF clusters 3 and 4 demonstrated visible RSN connectivity-matrix patterns (Figures 8A,B), IMF4 was associated with the highest signal power and highest correlation. The fact that IMF4 is also most likely to contain low-frequency physiological contributions supports previous findings that physiological processes are stable (Birn, 2012) and can equally generate highly robust connectivity-matrix patterns (Chang and Glover, 2009b). This is an important point to consider in interpreting the quality of RSN results based on strength and reproducibility alone.

However, our findings contrast previous findings that RSNs are a broadband phenomenon (Niazy et al., 2011; Qian et al., 2015). These previous findings are in line with band-pass filtering results (Figure 9), whereby similar connectivity patterns are observed across all frequency bands. Nonetheless, previous work has also demonstrated the frequency dependence of task-fMRI-based brain networks (Baria et al., 2011). Moreover, our results are also corroborated by near-infrared optical connectivity measures in the resting state (Sasai et al., 2011), whereby long-range and local connections were associated with distinct frequencies within the 0.009–0.1 Hz range.

As, we demonstrated significant mode mixing using EMD and CEEMD (Figure 1), we argue this effect could have resulted in the similarities between IMFs that were previously reported. The same logic may explain why band-pass filtered maps were similar across frequency bands, as IMFs are difficult to isolate using such filtering methods. Notwithstanding, the similarity of the IMF1 connectivity matrices obtained through VMD and CEEMD (Figure 8A) despite their different frequency bands, serves to cross validate previous findings against our findings at low frequencies. Lastly, we are unclear as to the reason the correlations values corresponding to VMD are much lower than those based on band-pass filtering (Figure 9).

On average (across the group), the functional connectivity values found with the VMD and CEEMD are lower than found with conventional bandpass filtering. This is to be expected, signal bands produced by BPF always have the same frequency ranges, but IMFs do not always have the same frequency ranges, and can vary from subject to subject in that regard. This variability can reflect in inter-subject variations in connectivity strength, as exemplified in the Appendix Figure A4.

### Limitations

In this work, we focused on the use of the empirical model decomposition (EMD) family of methods, and more specifically, on the use of the VMD method to provide estimates of intrinsic modes while avoiding mode mixing. In general, EMD has long been used for investigating the frequency composition of biological signals that are non-stationary. Compared to prevalent techniques such as independent-component analysis (ICA), EMD has the advantage of being able to operate on individual signals instead relying on multiple measurements. Furthermore, the focus on EMD is “intrinsic frequencies” instead of statistical independence, more directly addressing our main focus. Nevertheless, a combination of EMD and ICA may be investigated in our future work.

In adopting VMD, the main thrust of our work is to reduce the spectral overlapping in previous works. Our choice of IMFs is driven by precision and reproducibility, which may be a strength and a limitation, depending on whether the intrinsic modes are expected to be reproducible. Such assumptions have been used broadly in the rs-fMRI field, but requires further dissection. While the motivation for using VMD (and EMD in general) is the non-stationarity of the rs-fMRI signal, the ground-truth testing was done using simulated stationary signals. As it was unclear what alternative noise model would be appropriate for such a simulation (where the ground truth signal vs. noise distributions are unknown in rs-fMRI data), we used white noise. While this may be a limitation, such an approach provided us with a clear way to evaluate the techniques – if a given technique could not faithfully reconstruct a stationary signal, its performance on a non-stationary signal could be no better than presented. Although we have identified the frequency cluster IMF3 as most representative of neutrally relevant BOLD, both by frequency and by spatial contribution, we are not able, in the current study, to provide direct experimental verification. Likewise, we are unable to determine the amount of physiological contributions to IMF4, which is deemed most representative of respiratory and cardiac effects using the current data. In future studies, we will involve physiological monitoring during the rs-fMRI sessions. This will be augmented by the use of simultaneous EEG-fMRI to capture neural fluctuations as well as blood-oxygenation effects, ideally in the presence of stimuli that can modulate baseline cerebral metabolism. Furthermore, the use phase locking is also an effective tool for estimating the sources of the IMFs and their interplay (Pfurtscheller et al., 2017), given sufficient SNR.

Furthermore, while we determined that the cluster IMF2 is most likely associated with low-frequency vascular oscillations (or vasomotion), the central frequency of IMF2 is 0.15 Hz, deviating from the typically reported to be 0.1 Hz in surface vessels. Furthermore, we did not find any observable vascular networks based on specific frequencies in the fMRI signal. While we have evidence to believe the frequency of vasomotion increases with decreasing diameter (Intaglietta, 1990), our ability to isolate smaller blood vessels is limited by the spatial resolution of the fMRI acquisition and by the BOLD effect itself. One possibility for targeting this issue is to repeat these measurements in conjunction with independent monitoring of subcutaneous vasomotion as well as vascular stimuli that can modulate vasomotion amplitude.

Finally, in this study, we do not examine network properties such as the differences between local and long-range connections in our study of RSN frequency dependence. The intention of our current work is to establish the validity of our decomposition procedure, and a comprehensive examination of the frequency dependence of multiple network metrics will be part of our future work.

## Data Availability

The datasets generated for this study are available on request to the corresponding author.

## Ethics Statement

This study was approved by the Baycrest Research Ethics Board. All subjects provided informed written consent.

## Author Contributions

NY contributed to 90% of the analysis and 30% of the writing. NO contributed to 10% of the analysis. JC contributed to 70% of the analysis and writing.

## Conflict of Interest Statement

The authors declare that the research was conducted in the absence of any commercial or financial relationships that could be construed as a potential conflict of interest.

## Footnotes

1 http://surfer.nmr.mgh.harvard.edu
