# Supplementary material for: Intrinsic Frequencies of the Resting-State fMRI Signal: The Frequency Dependence of Functional Connectivity and the Effect of Mode Mixing
Source: Front Neurosci. 2019 Sep 4;13:900. doi: 10.3389/fnins.2019.00900 (PMC6738198; doi:10.3389/fnins.2019.00900)
Supplement: Supplementary file 1 [file Data_Sheet_1.docx]

**Appendix**

*The Assumption of Total Number of IMFs*

In applying VMD, a key assumption is the number of IMFs for the VMD algorithm to search for. As shown in Fig. A1, at an individual level, there is some dependence between the assumed IMF number and the IMF-clustering results.


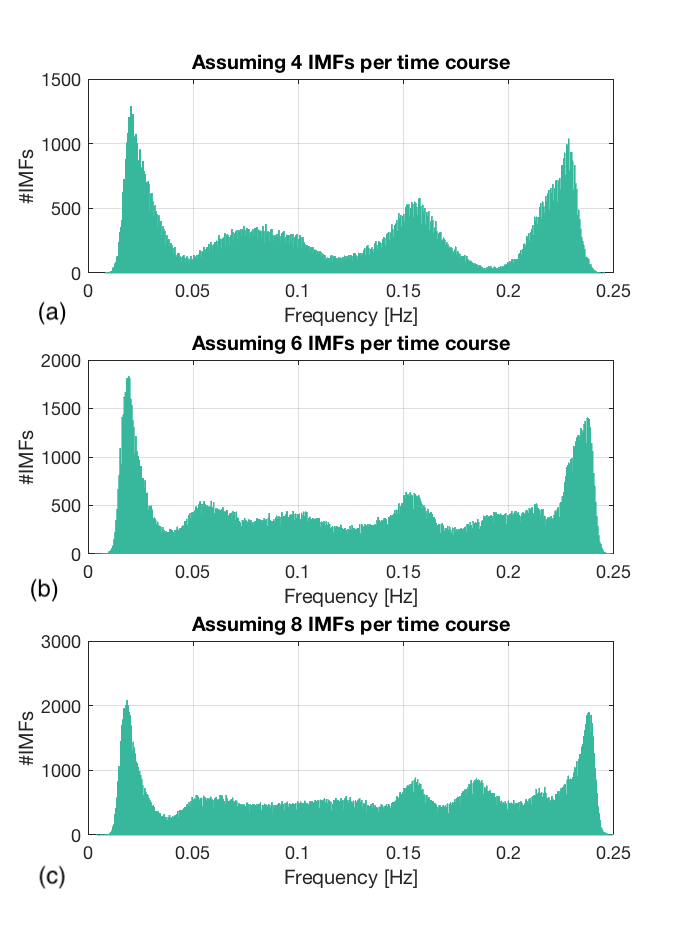


**Figure A1. The influence of assumed IMF numbers on the result VMD IMF histograms.** The histograms resulting from a sample dataset, vary slightly depending on the number of IMFs specified when running the VMD algorithm – 4 (a), 6 (b) and 8 (c). In this data set, an increasing number of assumed IMFs is shown to result in splitting of histogram peaks in the mid-frequency range (0.05-0.2 Hz). Nonetheless, the lowest and highest peaks are insensitive to the assumptions on the number of IMFs to fit for. In addition, it is important to note that the number of IMF-frequency clusters in our study is observed by examining all IMF histograms from all subjects and data sessions to identify the IMF clusters that are the most robust and consistent.

To assess the robustness of each individual assumption on the number of IMFs, we compared the IMF-clustering results based on assumptions of 2 IMFs, 4 IMFs, 5 IMFs and 8 IMFs. The results are shown in Figure A2, based on results from 2 sessions of rs-fMRI data with a TR of 2 s.

**
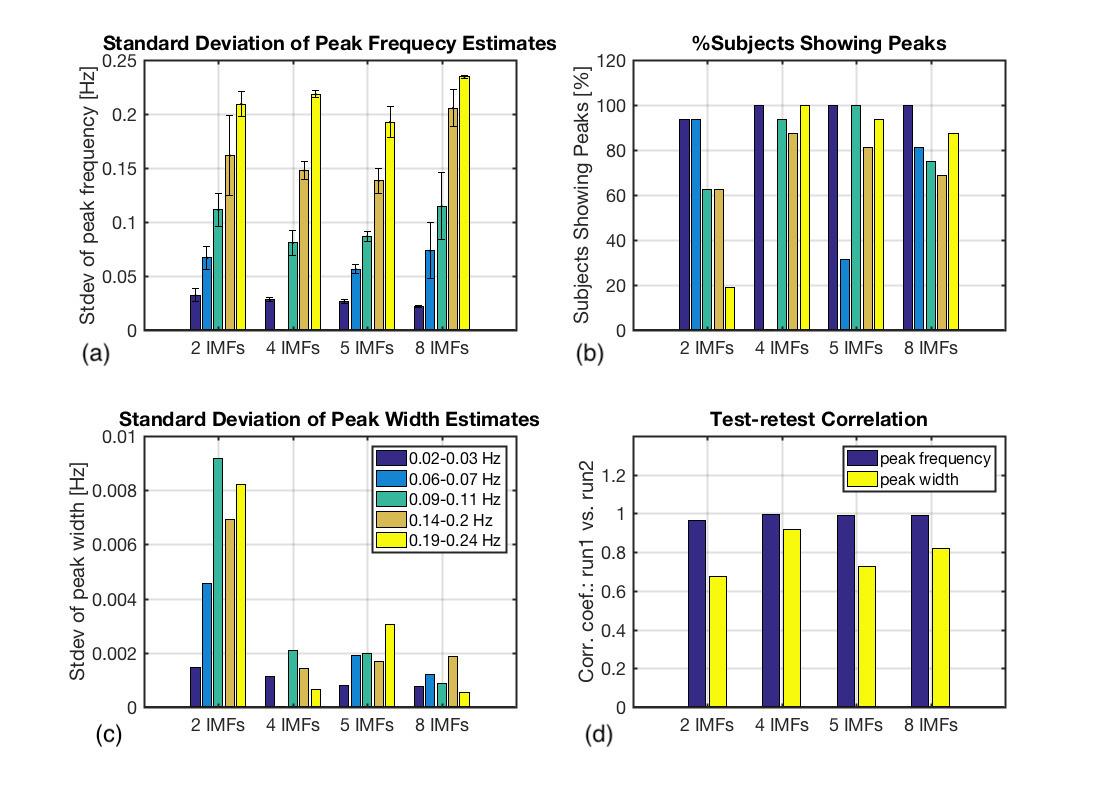
**

**Figure A2. Assessment of different assumptions regarding the number of IMFs in VMD.** (a) We targeted 2, 4, 5 and 8 IMFs, with all but 4-IMFs resulting in 5 IMF clusters. The errorbars represent inter-subject standard deviations. (b) Not all of the detected IMF clusters are reproducible across subjects, and only for the 4-IMF case are all of the resultant IMF clusters reproducible. (c) When targeting 4 IMFs, we also achieved the lowest inter-subject standard deviation in IMF-cluster widths. (d) The IMF cluster frequency and width are both highly reproducible only for the 4-IMF case in terms of the correlations between runs 1 and 2.

In the 4-IMF case, 4 IMF clusters are detected. In all other cases, 5 IMF clusters are detected (Fig. A2a). However, only some of these clusters are considered reproducible across subjects (being detected in >80% of all subjects and sessions, (Fig. A2b). Specifically, in the 4-IMF case, all resultant IMF clusters were highly reproducible across subjects and sessions (Fig. A2d). Moreover, the variability in the detected peak widths is also lowest in the 4-IMF case. Thus, targeting 4 IMFs resulted in the highest accuracy and reproducibility, and is adopted in our subsequent analyses.

*Spatial Variability in IMF-cluster Frequencies*

The IMF-cluster frequencies were found to be variable across brain regions, as shown in Fig. A3. The figure shows group-average regional-specific IMF-cluster frequencies in the grey matter, with IMF2 being the most spatially variable, while the other IMF-cluster frequencies were relatively consistent across regions.


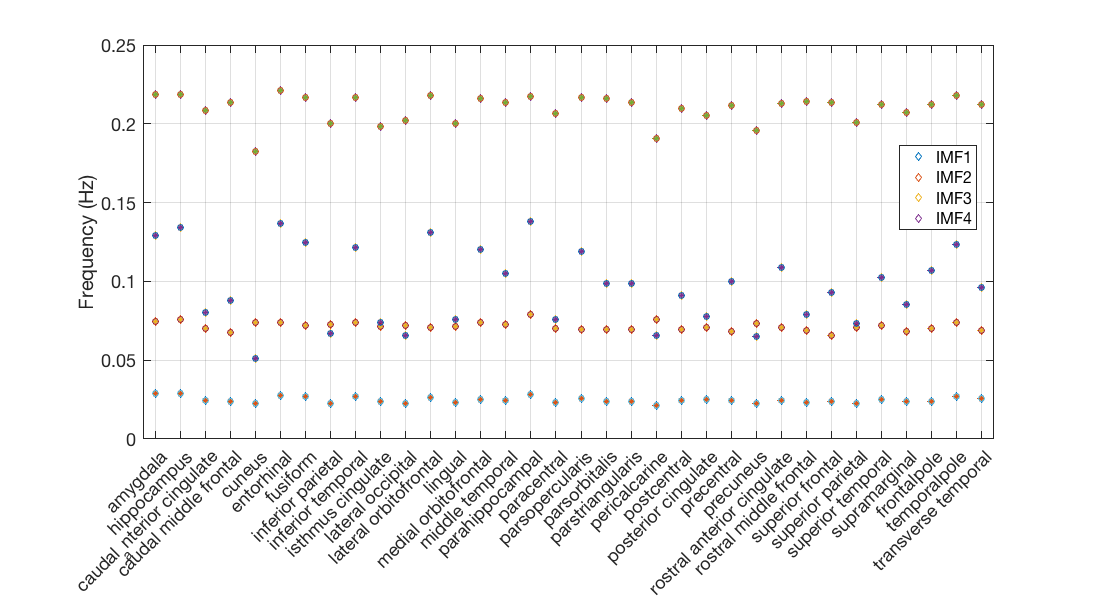


**Figure A3. Spatial variability in IMF-cluster peak frequencies.** IMF2 is the most spatially variable, while the other IMF-cluster frequencies were relatively consistent across regions. Notably, IMFs 3 and 4 are the most consistent across brain regions.

*Inter-subject Variability in Functional-connectivity Matrices*


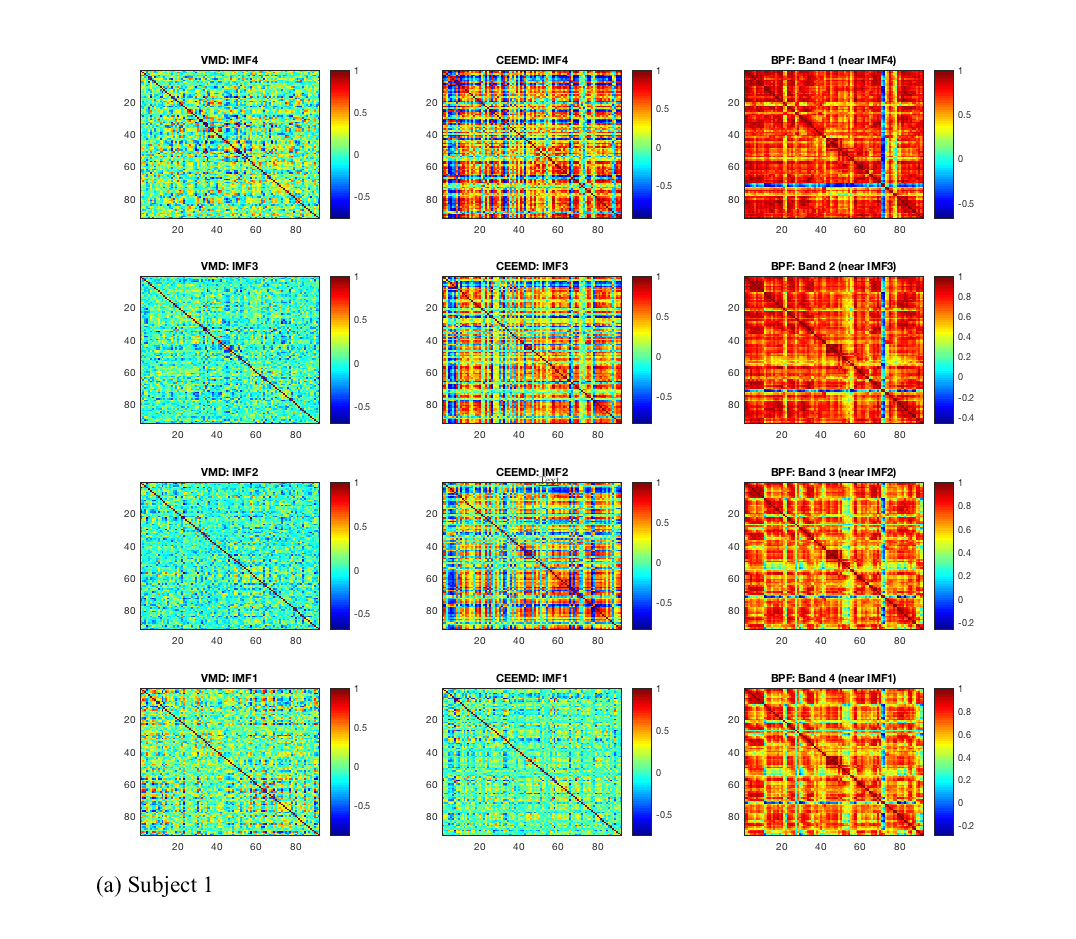


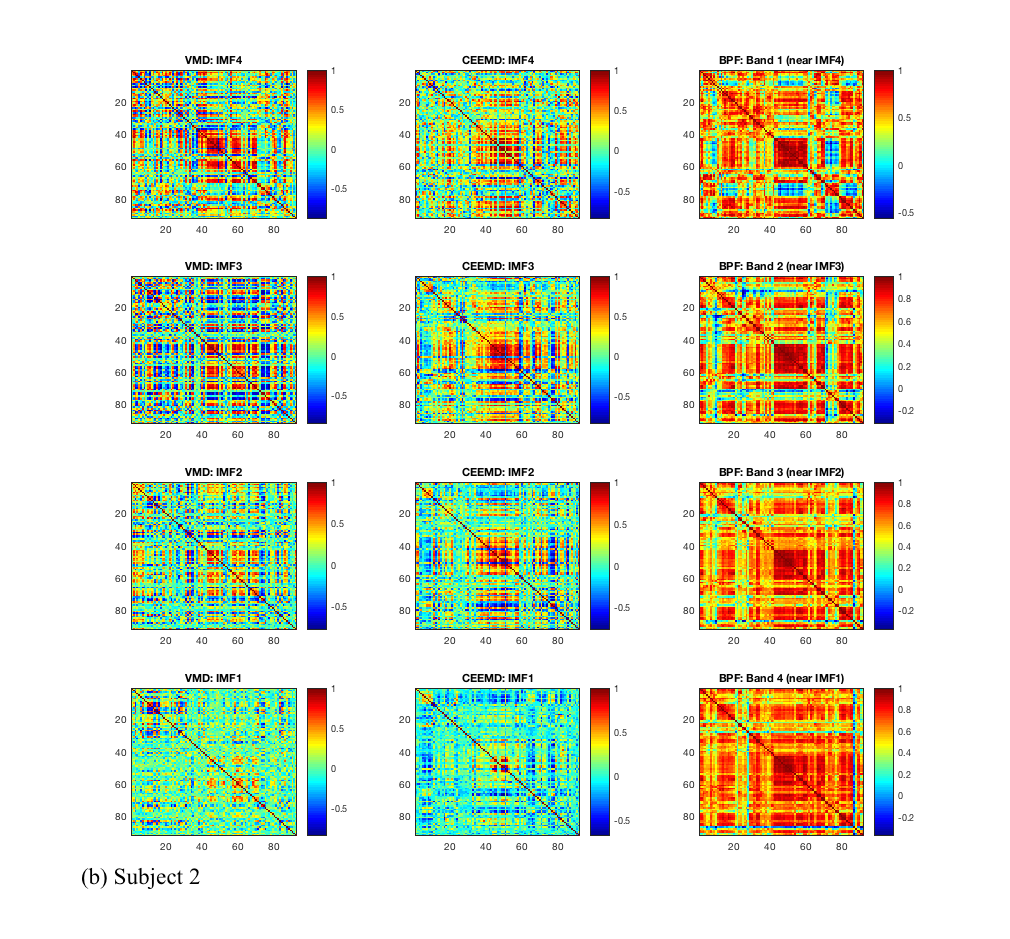


**Figure A4. Inter-subject variability in the functional-connectivity matrices obtained using VMD, CEEMD and band-pass filtering.** Correlation matrices are shown for two representative subjects (a and b). Both VMD and CEEMD yielded lower functional-connectivity scores than band-pass filtering (BPF) across all corresponding frequencies. However, this figure demonstrates considerable inter-subject variability in the EMD-based functional-connectivity values.
